# Epidemiological and Clinical Changes in Pediatric Acute Mastoiditis Before and After the COVID-19 Pandemic: An Eight-Year Retrospective Study from a Tertiary-Level Center

**DOI:** 10.3390/medsci13040297

**Published:** 2025-12-02

**Authors:** Marco Sarno, Antonia Pascarella, Antonietta De Lucia, Pietro Spennato, Fabio Savoia, Camilla Calì, Alida Casale, Adelia Dora, Giulia Meccariello, Raffaele Borrelli, Francesco Nunziata, Stefania De Caro, Emma Petrone, Iolanda Parente, Andrea Esposito, Camilla Russo, Eugenio Maria Covelli, Cristiana De Luca, Michele Schiavulli, Alessandro Perrella, Antonio della Volpe, Luigi Martemucci, Vincenzo Tipo, Paolo Siani, Giuseppe Cinalli

**Affiliations:** 1 Chronic Diseases, Hepatology and Nutrition Unit, Santobono-Pausilipon Children’s Hospital, AORN, 80129 Naples, Italy; m.sarno@santobonopausilipon.it (M.S.);; 2 Otolaryngology and Regional Referral Centre Pediatric Cochlear Implants, Santobono-Pausilipon Children’s Hospital, AORN, 80129 Naples, Italy; 3 Neurosurgery Unit, Department of Neurosciences, Santobono-Pausilipon Children’s Hospital, AORN, 80129 Naples, Italy; 4 Epidemiology, Biostatistics and Childhood Cancer Registry Unit, Santobono-Pausilipon Children’s Hospital, AORN, 80129 Naples, Italy; 5 Department of Translational Medical Sciences, Section of Pediatrics, University of Naples Federico II, 80138 Naples, Italy; 6 General Pediatrics and Immuno-Rheumatology Unit, Santobono-Pausilipon Children’s Hospital, AORN, 80129 Naples, Italy; 7 Pediatric Emergency Unit, Santobono-Pausilipon Children’s Hospital, AORN, 80129 Naples, Italy; 8 Neuroradiology Unit, Santobono-Pausilipon Children’s Hospital, AORN, 80129 Naples, Italy; 9 Clinical Pathology Unit, Santobono-Pausilipon Children’s Hospital, AORN, 80129 Naples, Italy; 10 Hemophilia and Congenital Bleeding Disorders Treatment Center, Santobono-Pausilipon Children Hospital, AORN, 80129 Naples, Italy; 11 Infectious Disease Division, PO Cotugno Hospital, AO dei Colli, 80131 Naples, Italy; 12 Chronic and Multifactorial Diseases Unit, Santobono-Pausilipon Children’s Hospital, AORN, 80129 Naples, Italy

**Keywords:** acute mastoiditis, intracranial complications, post-COVID-19 epidemiology

## Abstract

**Background**: Acute mastoiditis is the most frequent suppurative complication of acute otitis media in children. AM can lead to both extracranial complications and intracranial complications. Recent studies suggest an increase in cases after the COVID-19 pandemic. **Objective**: To compare the epidemiological and clinical characteristics of pediatric patients diagnosed with acute mastoiditis admitted to Santobono-Pausilipon Children’s Hospital before and after COVID-19. **Methods**: We conducted a retrospective study including all patients aged 0–16 years with AM admitted to our hospital between January 2017 and December 2024. Patients were stratified into three groups: pre-COVID-19: 1 January 2017–28 February 2020; COVID-19: 1 March 2020–31 December 2021; and post-COVID-19: 1 January 2022–31 December 2024. Demographic data, clinical presentations, complications, laboratory findings, and treatment modalities were analyzed and compared between groups. **Results**: A total of 276 children (153 males and 123 females; median age: 49 months, age range: 1–177 months) were included. Hospital admissions for AM increased in the post-COVID-19 period, reaching more than a threefold increase in 2024 compared with the pre-COVID-19 years. Similar to the overall number of AM cases, the absolute number of complications, especially IC, such as thrombosis and empyema, increased. The rate of surgical procedures increased during the post-COVID-19 period, with an overall increase of 88.5%. Both the duration of antibiotic therapy and hospital stay were significantly longer in the post-COVID-19 period. **Conclusions**: The COVID-19 pandemic has been associated with epidemiological and clinical changes in pediatric AM patients. These findings highlight the need for effective preventive strategies, including enhanced vaccination coverage and the promotion of early diagnosis. Additionally, implementing standardized clinical protocols could support more efficient and consistent management, reducing hospital stays and recurrence rates.

## 1. Introduction

Acute mastoiditis (AM) is the most common suppurative complication of acute otitis media (AOM) and its incidence varies across different reports and countries, ranging from 1.2–6.1 per 100,000 patients per year in children aged 0–14 years [1,2,3]. The epidemiology of AM closely mirrors that of AOM, with the highest incidence observed in children under two years of age, generally by the age of four. The diagnosis of AM is primarily clinical and is typically made in the context of a recent or ongoing episode of AOM. Laboratory tests typically show signs of systemic inflammation, such as leukocytosis and elevated inflammatory markers, including C-reactive protein (CRP) [4]. Up to 50% of AM episodes occur during the first episode of AOM. The overall incidence of AM requiring surgical intervention is 0.004% in the United States (US), with a current incidence likely under 2 per 100,000 people, although varying numbers are present in the literature [5]. AM can lead to both extracranial complications (ECs), such as subperiosteal abscess, facial nerve paralysis, hearing loss, labyrinthitis, osteomyelitis, and Bezold abscess, and intracranial complications (ICs), such as meningitis, temporal lobe or cerebellar abscess, epidural or subdural empyema, and venous sinus thrombosis. The rate of complications of AM is approximately 15–30% (range, 7–60%) [6]. The most frequently reported type of EC is subperiosteal abscess [7], whereas the most common type of IC is epidural or extradural empyema [8]. Venous sinus thrombosis among IC patients is certainly the most feared, with a reported frequency of 2.7% in retrospective studies [9,10]. *Streptococcus pneumoniae* (*S. pneumoniae*) and *Streptococcus pyogenes* (*S. pyogenes*) are the most common causative agents of AM; however, nontypeable *Haemophilus influenzae* (*H. influenzae*), *Staphylococcus aureus* (*S. aureus*), *Pseudomonas aeruginosa* (*P. aeruginosa*) and anaerobic bacteria have been isolated [11]. Imaging is crucial for identifying severe complications. However, the optimal timing of imaging is debated, and the available evidence does not allow for a clear indication. The use of computed tomography (CT) scans in the diagnostic process is still a controversial issue today: its examination rate greatly differs among different papers, varying from 0.4% to 100%. While some authors recommend imaging only when complications are suspected to minimize radiation exposure, others suggest early imaging to promptly identify and manage complications. According to a 2025 systematic review, the diagnosis of AM was based exclusively on clinical findings in 60.8% of cases [12]. The current gold standard is cranial magnetic resonance imaging (MRI); however, there is still a lack of availability, especially in urgent cases [13]. Microbiological identification of the causative pathogen is crucial, particularly in cases where antibiotic resistance is suspected or the clinical course is atypical. The variability in pathogen isolation rates reported across different case series is likely influenced by the administration of antimicrobial therapy prior to microbiological sampling, which is often initiated for the treatment of antecedent acute otitis media. Pretreatment with antibiotics can reduce the sensitivity of culture-based diagnostic methods, thereby impacting the detection and accurate identification of causative organisms [14]. The management of AM is determined by the severity of the clinical presentation and the presence of local or systemic complications. Treatment typically involves a combination of parenteral antimicrobial therapy and surgical intervention, the latter primarily performed by otolaryngologists and, in selected cases with intracranial involvement, in collaboration with neurosurgeons. Currently, there is a lack of standardized, universally accepted protocols guiding the medical and surgical treatment of AM. Surgery seems to be the most effective treatment for complicated AM with respect to medical and conservative management [15]. The treatment of AM has changed during recent decades, shifting to more conservative management, even if there is still great variability in practice. AM are generally treated via intravenous (IV) antibiotics when available and via myringotomy with or without tube placement. To date, there is no evidence to guarantee the efficacy and safety of oral therapy as a first-line treatment, even in uncomplicated cases. Given the prevalence of *S. pneumoniae* and its particular sensitivity to cephalosporins, ceftriaxone is frequently employed first-line, with ampicillin-sulbactam being an appropriate alternative. Vancomycin is reserved for severe cases, treatment failure or suspected complications and is not a first-line treatment because of the low prevalence of methicillin-resistant *S. aureus*. The transition to oral antibiotics is effective once clinical improvement and culture results are confirmed, with longer courses (4 weeks) for intracranial complications. On the basis of the available data in the literature, the duration of antibiotic therapy is not standardized and ranges from 4–30 days [16,17]. Many studies have also shown that antibiotics alone may be adequate in most uncomplicated cases [18]. Data reported in the literature regarding the incidence of uncomplicated or complicated forms of AM in the pediatric population primarily refer to the pre-COVID-19 period. The COVID-19 pandemic strongly changed the epidemiology of upper respiratory tract infections in pediatric patients. Nonpharmaceutical interventions (NPIs), such as physical distancing, face masks and societal behavior, led to a sharp decrease in non-SARS-CoV-2 infections during the pandemic period [19]. Distancing measures minimize exposure to common pathogens and reduce early childhood immunization, leading to changes in the epidemiology and course of respiratory infections. When restriction measures are reduced, modifications in the seasonal circulation patterns of endemic viral diseases and an increase in pediatric cases of pneumonia caused by respiratory syncytial virus (RSV), influenza, and *Mycoplasma pneumoniae* have been reported [20,21,22,23].

During the first 2 years of the US COVID-19 pandemic, pediatric centers anecdotally reported increased rates of intracranial bacterial infections, many of which were diagnosed during or immediately after an infection with SARS-CoV-2 [24]. In 2022, the Emergency Infections Network recruited 8 centers in the U.S. to study the trends of streptococcal-related sinusitis and AM during the pre-COVID-19 period (January 2018–January 2020) and the COVID-19 period (March 2020–March 2022) in children. The results revealed a significant increase in intracranial infections during the COVID-19 period and sinusitis complicated by intracranial infections, along with a decrease in orbital cellulitis, sinusitis, mastoiditis and mastoiditis complicated by intracranial infection. The partial discrepancy between these data could be explained by recruitment biases, the small number of participating centers, voluntary participation in the study, the limited data collected and the lack of details about the patients [25]. Following this evidence, the European Society of Pediatric Neurosurgery requested that its members from 14 different European countries submit clinical and radiological data on all cases of IC associated with sinusitis and AOM. The cases were divided into three periods: January 2017–December 2019 (pre-COVID-19), January 2020–December 2021 (COVID-19), and January 2022–June 2023 (post-COVID-19). A total of 254 cases were collected and analyzed from 31 European centers. A significant difference was found in the IC of sinusitis and AOM between the pre-COVID-19 period (85 cases) and the COVID-19/post-COVID-19 period (169 cases) (*p* = 0.001), as well as in the annual incidence of cases (28.3 vs. 20 vs. 86, *p* = 0.00) [26]. With this background, the aim of our study was to investigate the incidence of uncomplicated and complicated forms of AM in a cohort of pediatric patients hospitalized due to AM in the pre-COVID-19 period and post-COVID-19 period and to analyze any differences in the characteristics of the two groups.

## 2. Materials and Methods

We conducted an 8-year retrospective study (2017–2024) on pediatric patients diagnosed with AM admitted to Santobono-Pausilipon Children’s Hospital. This institution is the main tertiary pediatric referral center in southern Italy and a reference center for pediatric neurosurgical and otorhinolaryngological conditions. Patients under 16 years of age admitted between 1 January 2017, and 31 December 2024, to the Departments of Pediatrics, Neurosurgery, and Otorhinolaryngology were considered eligible for inclusion. Case identification was performed using ICD-9-CM discharge diagnosis codes extracted from hospital discharge records. For all identified admissions, medical charts were reviewed in detail. The diagnosis of AM was established on the basis of a combination of clinical findings and radiological imaging (CT and/or MRI). Specifically, clinical signs suggestive of AM include retroauricular edema and erythema, flattening of the retroauricular groove, and auricular protrusion, which are associated with features of acute otitis media, such as tympanic membrane alterations, otalgia, and otorrhea.

CT was performed upon medical judgment (pediatrician and/or otorhinolaryngologist and/or neurosurgeon) in cases of clinical suspicion of AM, both in the Emergency Department and in hospital wards. MRI was performed in selected patients who, on CT, presented either IC or EC complications on the basis of clinical assessment after consultation with a neuroradiologist.

Posttraumatic cases were excluded. For each enrolled patient, the following demographic and clinical data were collected: sex, age, length of hospital stay, presence of comorbidities, clinical signs and symptoms at presentation, any prior outpatient treatments (including antibiotic therapy and/or nonsteroidal anti-inflammatory drugs (NSAIDs), vaccination status for *H. influenzae* and *S. pneumoniae*, laboratory results (inflammatory markers and leukocyte count), radiological investigations (CT/MRI), available microbiological data, type and duration of antibiotic therapy, need for surgical intervention and type of procedure performed, presence of ICs and/or ECs, and requirement for anticoagulant therapy. We used PCT and PCR cutoff values higher than 0.5 ng/mL and 20 mg/L, respectively. These cutoffs are optimal for detecting invasive and serious bacterial infections [27,28]. Leukocytosis was defined as white blood cell counts above the upper age-specific reference limit [29]. The primary objective of the study was to describe hospital admissions for AM in the pediatric population and to assess temporal trends in incidence, clinical features, occurrence of complications, and therapeutic approaches (both medical and surgical). To this end, hospitalizations were categorized into three time periods in relation to the COVID-19 pandemic: the pre-COVID-19 period: 1 January 2017–28 February 2020; the COVID-19 period: 1 March 2020–31 December 2021; and the post-COVID-19 period: 1 January 2022–31 December 2024. However, some of the comparative analyses were conducted by restricting the comparison to the pre-COVID-19 period (used as the reference) and the post-COVID-19 period. Further subgroup analyses were conducted to compare patients with AM in relation to surgical intervention (need and type), complications, and the duration and type of antibiotic therapy.

### Statistical Analysis

Hospital admissions for AM were reported as absolute and relative frequencies to describe temporal variations across the study period. Incidence rate ratios (IRRs) with 95% confidence intervals (CIs) were estimated via Poisson regression models on the basis of the number of new admissions for AM and for the main IC and EC observed in the post–COVID-19 period, with the pre–COVID-19 period as the reference. Associations between demographic, clinical, and therapeutic variables and both the hospitalization period and the occurrence of complications were assessed. Categorical variables were compared via the chi-square test or Fisher’s exact test, when appropriate. For continuous variables, expressed as medians and interquartile ranges (IQRs), nonparametric comparisons were performed via the Mann–Whitney U test, which is a test of the equality of medians between groups.

## 3. Results

When the inclusion and exclusion criteria were applied to the subjects identified through the review of medical records, a total of 276 patients diagnosed with AM were included in the study (153 males and 123 females; median age: 49 months, age range: 1–177 months).

**Figure 1:**
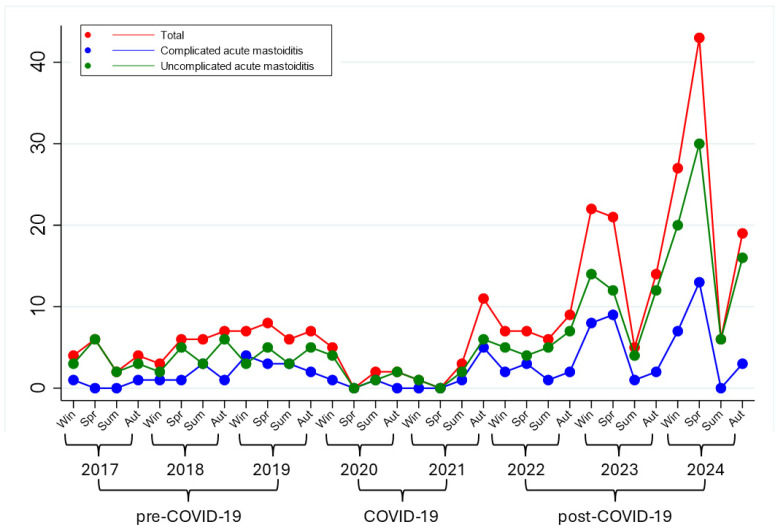
Temporal trend of hospital admissions for AM forms across the pre-COVID-19, COVID-19, and post-COVID-19 periods.

During the period of 2017–2024, an average of 34.5 hospitalizations per year for AM were recorded, with marked temporal variations. During the pre–COVID-19 period (2017–February 2020), the mean incidence was 22.2 cases/year (70 cases in total). During the COVID-19 period (March 2020–December 2021), hospitalizations were almost halved, with an average of 10.9 cases/year. From 2022 onward, however, a clear increase was observed. In the post–COVID-19 period (2022–2024), the mean number of cases increased to 61.9 cases/year, corresponding to a 2.8-fold increase compared with that in the pre–COVID-19 period. This increase was not uniform across the three years but progressively intensified: compared with the prepandemic reference, hospitalizations increased by 32% in 2022, by 82% in 2023, and reached more than a fivefold increase in 2024 (Figure 1).

### 3.1. Patient Characteristics

Among the 276 enrolled patients, 7 presented with associated chronic conditions: 1 case of congenital cytomegalovirus infection in the pre-COVID-19 period, 1 case of Becker syndrome in the COVID-19 period, 1 case of Duane syndrome, 1 case of Klippel-Feil syndrome, 2 cases of Down syndrome, and 1 congenital disorder of glycosylation in the post-COVID-19 period. Only one patient, enrolled during the post-COVID-19 period, had primary immunodeficiency (DiGeorge syndrome).

A known predisposing factor for AM was identified in 13 out of 276 patients: 1 case of cholesteatomatous otitis media in the pre-COVID-19 period, 1 case of chronic otitis media in the COVID-19 period, and 1 case of recurrent otitis media and 10 cases of adenoidal hypertrophy in the post-COVID-19 period.

No statistically significant differences were observed in sex distribution across the time periods (pre-COVID-19: 51.4% male, 48.6% female; post-COVID-19: 55.9% male, 44.1% female; *p* value = 0.26). The median age at admission did not differ significantly across time periods (in the pre-COVID-19 period: median 55 months (IQR 30–87), in the post-COVID-19 period: median 48 months (IQR 26–77) (*p* value = 0.26)). Antibiotic treatment prior to hospital admission was reported in 50.7% of patients, with no significant difference between time periods (*p* value = 0.77). However, prehospital antibiotic use was significantly associated with complicated AM (*p* < 0.05), which was reported in 63% of complicated cases compared with 46% of noncomplicated cases. Data on prior antibiotic use were unavailable for 36 patients. NSAIDs were administered at home in the majority of cases: 112 out of 208 patients for whom data were available. No association was found between NSAIDs use and complicated AM (*p* value = 0.23). However, NSAIDs use was significantly more common during the post-COVID-19 period than during the pre-COVID-19 period (65.4% vs. 10.3%, *p* value < 0.01). During the COVID-19 period, 4 out of 20 (20%) patients used NSAIDs prior to hospitalization. Information on the duration and dosage of NSAIDs was not available or complete.

Seventy percent of patients enrolled in the pre-COVID-19 period and 63% of patients enrolled in the post-COVID-19 period received the anti-*Haemophilus influenzae* and anti-pneumococcal vaccines. Vaccination status data were unavailable for 30% of pre-COVID-19 cases and 37% of post-COVID-19 cases. During the COVID-19 period, 16 out of 20 patients received the anti-*Haemophilus influenzae* and antipneumococcal vaccines; for 3 patients, this information was not available. Vaccination was considered “protective” when the patient had received at least two doses. Patients who had not yet started the vaccination schedule or had received only one dose were considered unvaccinated. Data regarding the type of pneumococcal vaccine administered (7-valent or 13-valent) are unreliable. Specifically, in children previously vaccinated with the 7-valent pneumococcal vaccine, the administration of a catch-up dose with the 13-valent vaccine has not been recorded.

A comparative analysis of presenting signs and symptoms of AM between the pre-COVID-19 and post-COVID-19 periods revealed statistically significant differences (*p* < 0.05) in the frequency of headache, fever, vomiting, altered consciousness, and cranial nerve deficits (Table 1).

### 3.2. Complications of AM

Complications were identified in 28.6% (*n* = 79) of patients hospitalized for AM. The proportion of complicated forms did not significantly vary over time, with no statistically significant differences among the three analyzed periods. During the pre-COVID-19 period, 70 patients were admitted with AM, 20 (28.6%) of whom developed complications. During the COVID-19 period, 8 out of 20 patients (40%) had complicated AM, whereas during the post-COVID-19 period, 51 out of 186 patients (27.4%) experienced complications (*p* value = 0.50).

Patients with complicated AM differed from those with uncomplicated AM in terms of age at admission. The median age for complicated cases was 55 months (IQR 39–89), whereas it was 45 months (IQR 25–71) for uncomplicated cases (*p* value < 0.01).

Similar to overall AM cases, the absolute number of complicated cases of AM increased over time, increasing from an average of 6.6 admissions per year in the pre-COVID-19 period to 16.7 per year in the post-COVID-19 period.

A total of 140 complications were identified in 79 patients. Among these patients, 53 (67%) had at least one IC, 40 (51%) had at least one EC, and 14 (18%) had both. The most frequent CIs included thrombosis (40.8%, 38 patients) and epidural empyema (29.0%, 27 patients). The most common type of EC was subperiosteal abscess, accounting for 30.1% (28 cases) of all complications.

When comparing the pre-COVID-19 and post-COVID-19 periods, a significant increase in the incidence of IC was observed, with a mean annual frequency of 12.3 patients, which was more than three times greater than that in the pre-COVID-19 period (IRR 3.25; 95% CI: 1.66–6.86). Among ICs, a statistically significant increase was noted in both intracranial thromboses (IRR 3.05; 95% CI: 1.38–7.39) and epidural empyema (IRR 2.71; 95% CI: 1.08–7.68). Although an increase in the number of ECs was observed in the post-COVID-19 period, this increase did not reach statistical significance (IRR 1.51; 95% CI: 0.72–3.23) (Table 2).

### 3.3. Laboratory Findings and Radiological Investigations

Radiological confirmation of AM through cranial CT or MRI was obtained in 229 out of 276 patients (83.0%).

All 229 patients underwent CT, 65 of whom also underwent MRI. Among the 229 patients who underwent neuroimaging, 150 (65.5%) had uncomplicated forms of AM, whereas 79 had complicated forms. Sixteen (10.7%) of the 150 patients with uncomplicated AM also underwent MRI. Among the 79 patients with complicated AM, 49 (62%) also underwent MRI. In the pre-COVID-19 period, 46 out of 70 patients (65.7%) underwent neuroimaging, and among them, 9 (19.6%) also underwent MRI. During the COVID-19 period, 16 out of 20 patients (80%) underwent neuroimaging, of whom 5 (31.2%) had MRI.

In the post–COVID-19 period, 167 out of 186 patients (89.8%) underwent neuroimaging, of whom 51 (30.5%) had MRI. Although there was increased use of MRI in the post–COVID-19 period, this increase was not statistically significant compared with that in the pre–COVID-19 period. (*p* value = 0.14).

CRP levels were measured in 272 patients (98.6%). Elevated CRP values (>20 mg/L) were observed in 189 patients (69.5%). In particular, the CRP level was significantly elevated in 128 out of 195 patients with uncomplicated AM (65.6%) and in 61 out of 77 patients with complicated AM (79.2%) (*p* value < 0.05). The median CRP level was significantly greater in patients with complicated AM (101.1 mg/L, interquartile range [IQR] 56.2–110.0) than in those with uncomplicated AM (67.3 mg/L, IQR 42.7–126.0), with a *p* value < 0.05.

PCT was measured in 198 patients (71.7%), including 61 patients with complicated AM and 137 patients with uncomplicated AM. A positive PCT value (>0.5 ng/mL) was observed in 40.9% of complicated cases and in 23.4% of uncomplicated cases, indicating a statistically significant association with disease severity (*p* < 0.05). Similarly, when only patients with positive PCT values were considered, the median PCT level was greater in complicated AMs (1.85 ng/mL, IQR 1.1–11.7) than in uncomplicated AMs (1.40 ng/mL, IQR 1.0–2.2), although the difference did not reach statistical significance (*p* = 0.15).

Leukocyte counts were available for the entire cohort. Leukocytosis was present in 52 out of the 79 patients with complicated AM (65.8%) and in 116 out of the 197 patients with uncomplicated AM (58.8%). Although leukocytosis was more common in complicated patients, the difference in leukocytosis rates did not reach statistical significance.

### 3.4. Surgical Management

A total of 131 patients out of 276 (47.4%) underwent 148 surgical procedures, including both complicated and uncomplicated forms of AM. Among patients with complicated AM, 10 out of 79 (12.6%) required more than one surgical intervention during hospitalization. The surgical procedures performed included tympanic membrane paracentesis, mastoidectomy, craniotomy, cerebrospinal fluid drainage, and other procedures (including external ventricular drainage and drainage of retroauricular collections).

A total of 41 procedures were performed in the pre-COVID-19 period, whereas 98 procedures were performed in the post-COVID-19 period, corresponding to an overall increase of 88.5%. Mastoidectomy and tympanic paracentesis were the most frequently performed procedures in both periods, accounting for 25.7% and 24.3%, respectively, of patients hospitalized for AM in the pre-COVID-19 period and 22.0% each in the post-COVID-19 period. Other surgical procedures included craniotomy (2 patients in the pre-COVID-19 period and 6 in the post-COVID-19 period), CSF drainage (1 in the pre-COVID-19 period and 6 in the post-COVID-19 period), and other interventions (3 in the pre-COVID-19 period and 4 in the post-COVID-19 period). The distribution of procedures was similar in the two periods, with no statistically significant differences between intervention types (*p* > 0.05).

Among the 276 patients, three experienced disease recurrence, all of whom were observed during the post-COVID-19 period.

### 3.5. Microbiological Data

Intraoperative cultures were obtained from 68 out of 148 surgical cases (52.7%). Among these, 27 samples (39.7%) tested positive. The most frequently isolated pathogens included *S. pyogenes* (*n* = 9), *S. pneumoniae* (*n* = 3), *S. aureus* (*n* = 1), coagulase-negative staphylococci (CoNS; *n* = 5), other streptococci (*n* = 2), anaerobic bacteria (*n* = 4), *Escherichia* coli (*E. coli*, *n* = 1), fungi (*n* = 1), and one other unspecified organism. Among the 27 culture-positive cases, 19 (70.4%) were classified as complicated. Among the isolates, 1 out of 9 *S. pyogenes* strains (11.1%) was resistant to clindamycin, and 3 out of 9 (33%) were resistant to quinolones. All *S. pyogenes* isolates remained susceptible to beta-lactams, as expected. Two additional nonpyogenic streptococci were also susceptible to beta-lactams. Among the *S. pneumoniae* isolates, 1 out of 3 (33%) was resistant to cephalosporins. Furthermore, *S. aureus* was susceptible to oxacillin, and *E. coli* was susceptible to beta-lactams combined with beta-lactamase inhibitors, as were cephalosporins and quinolones. Finally, 3 out of 5 (60%) coagulase-negative staphylococci (CoNS) were resistant to oxacillin.

### 3.6. Antibiotic and Anticoagulation Therapy

IV antibiotic therapy was administered to 272 out of 276 patients (98.6%). The four patients who did not receive IV antibiotics had uncomplicated forms. The most frequently used antibiotic was ceftriaxone, which was prescribed to 229 of the 272 patients. Its use was particularly common in the post-COVID-19 period, where it was administered in 154 out of 186 cases. Combination antibiotic therapy involving at least two agents was employed in 101 patients (37.1%). The most frequently used combinations were ceftriaxone with clindamycin (41 patients) and ceftriaxone with vancomycin (9 patients). Meropenem, either as monotherapy or in combination, was used in 25 patients.

Anticoagulant therapy was administered to 29 patients, all of whom had documented intracranial thrombosis. Anticoagulant therapy was administered to 29 patients, all of whom had documented intracranial thrombosis. Enoxaparin was used in all the patients. Five of these patients were treated during the pre-COVID-19 period, and 24 were treated during the post-COVID-19 period.

### 3.7. Duration of Therapy and Length of Hospitalization

**Figure 2:**
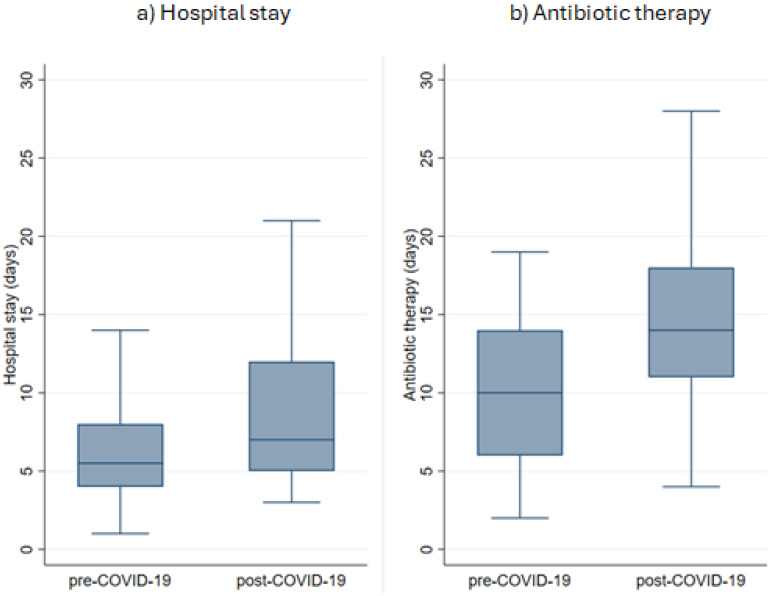
Distribution of hospital stay duration (**a**) and antibiotic therapy (**b**) between the pre-COVID-19 period and the post-COVID-19 period.

The total duration of antibiotic therapy was significantly longer in patients with complicated forms, with a median of 21 days (IQR 13–32), than in those with noncomplicated forms (IQR 10–14) (*p* < 0.01). A similar trend was observed for IV antibiotic therapy, with a median of 14 days (IQR 8–25) in complicated cases and 5 days (IQR 4–7) in uncomplicated cases (*p* value < 0.05). When the pre- and post-COVID-19 periods were compared, there was a significantly longer duration of antibiotic therapy in the post-COVID-19 group (median 14 days, IQR 11–18) than in the pre-COVID-19 group (median 10 days, IQR 6–14; *p* < 0.05). Consistently, the duration of IV antibiotic therapy was longer in the post-COVID-19 group (median 7 days, IQR 5–12) than in the pre-COVID-19 group (median 5 days, IQR 4–7) (*p* value < 0.05). The duration of hospital stay was longer in the post-COVID-19 period, with a median of 7 days (IQR 5–12) versus 5.5 days (IQR 4–8) in the pre-COVID-19 period (*p* < 0.01). The duration of hospitalization was also significantly longer in patients with complicated forms, with a median stay of 14 days, than in patients with noncomplicated cases, with a median stay of 5 days (*p* < 0.01) (Figure 2).

## 4. Discussion

The COVID-19 pandemic has significantly impacted the epidemiology and clinical presentation of infectious diseases across all age groups. Several studies have reported alterations in the incidence, severity, and microbial profile of both viral and bacterial infections during and after the pandemic [30,31]. During the 2020–2021 COVID-19 period, a noticeable decrease in AOM and subsequent AM was observed [32].

In this context, the changes observed in the epidemiology of pediatric AMs are consistent with patterns described in other pediatric bacterial infections. Starting in the fall of 2022, an increase in pediatric invasive *S. pyogenes* cases was reported in the U.S. compared with the pre-COVID-19 period. This increase coincided with a rise following an early and record-breaking surge in hospitalizations for RSV and influenza [33].

Moreover, in the United Kingdom and other regional European countries, such as Ireland, France, the Netherlands, and Spain, there has been a reported increase in cases of noninvasive *S. pyogenes* infections (fever and scarlet) and invasive *S. pyogenes* infections and related deaths, primarily among children under 10 years of age [34].

Several hypotheses have been proposed to explain the observed shifts in epidemiological patterns, including the altered course of endemic viral infections, changes in bacterial disease dynamics in pediatric populations, and the increase in invasive infections following the COVID-19 pandemic. NPIs, while effective in curbing the transmission of SARS-CoV-2 and other viral and bacterial pathogens, confer only short-term benefits. As early as 2021, concerns emerged regarding the unintended consequences of these public health measures. Reduced exposure to common pathogens and the associated lack of immune stimulation, coupled with a decline in routine vaccination coverage, contributed to what has been termed an “immunological debt.” This phenomenon, particularly among preschool-aged children, likely increases the number of susceptible individuals and diminishes herd immunity [35,36].

Disruptions to regular healthcare visits during the pandemic resulted in delays in immunization, leaving some children unprotected against vaccine-preventable bacterial diseases such as pneumococcal infections, *H. influenzae* type b infections and influenza [37]. This has been confirmed by numerous studies, including both modeling projections and epidemiological analyses conducted by leading infectious disease surveillance organizations, such as the European Centre for Disease Prevention and Control [38,39,40].

Our retrospective study highlighted significant changes in the epidemiology of pediatric AM during the pre- and post-COVID-19 periods. In particular, we observed a marked decrease in hospital admissions during the pandemic phase (March 2020–December 2021), which was likely related to containment measures and reduced access to nonurgent healthcare services. However, starting in 2022, there was a progressive and substantial increase in cases, peaking in 2024, when hospitalizations nearly tripled compared with those in the pre-COVID-19 period. This trend may reflect a rebound effect due to decreased exposure to common pathogens during the previous two years, when stricter measures were adopted, resulting in reduced herd immunity and increased susceptibility to infection. This increase was accompanied by a significant increase in IC, particularly sigmoid sinus thrombosis and epidural and subdural empyema, with a threefold higher incidence than that in the pre-COVID-19 period.

During the COVID-19 pandemic, access to hospital care was markedly restricted due to concerns about viral exposure, leading to a substantial decline in pediatric emergency department visits and delays in seeking care. This likely accounts for the overall reduction in the incidence of AM observed during the lockdown period. Although the overall incidence decreased, in our series, the rate of complications appeared to increase during the COVID-19 period, likely reflecting that only the most severe cases presented to hospital care. Nevertheless, the absolute number of AM cases, particularly complicated forms, during the COVID-19 period was too limited to allow for meaningful statistical analysis.

In our study, the inferential analysis specifically focused on comparing the pre–COVID-19 and post–COVID-19 periods to highlight possible epidemiological and clinical changes in AM after the pandemic, using the pre–COVID-19 period as a background reference. In addition, the limited number of cases during the COVID-19 phase would have reduced the statistical power and the ability to detect meaningful differences across three times intervals; therefore, this period was not included in inferential comparisons. For these reasons, we maintained the three-period framework in the descriptive analyses but restricted inferential comparisons to the pre- and post–COVID-19 periods.

In line with our findings, a recent retrospective study of 75 pediatric patients reported an increased incidence of AM cases requiring mastoidectomy, as well as a higher rate of complicated forms in the post-COVID-19 period [41].

In contrast to what has been reported in the literature, in our case series, the average age of patients with AM was greater (median 49 months). Interestingly, in our case series, patients with a history of acute or chronic otitis media did not present a higher rate of complicated forms. Instead, prior antibiotic use was found to be an important factor linked to the development of IC. It may be hypothesized that the home use of antibiotics before hospitalization was suboptimal with respect to both dosage and duration. A recent retrospective study of 298 patients also highlighted similar findings, comparing cases of am with and without IC [42].

Analysis of clinical features revealed a significant increase in the frequency of symptoms such as headache, vomiting, and altered mental status in the post-COVID-19 period. This increase may be partly attributed to greater infection severity, possibly linked to delays in diagnosis.

Although limited, our microbiological data indicate the continued predominance of classic pathogens such as *S. pyogenes* and *S. pneumoniae*, with no evidence of significant changes in the responsible flora. We had a high percentage of positive cultures for coagulase-negative staphylococcus, but we cannot rule out that it may be a skin contaminant. We also detected a high percentage of negative cultures, which was probably due to antibiotic therapy administered prior to hospitalization. The low number of isolates does not allow for definitive recommendations on empirical therapy. However, at least among our isolates, there is a favorable susceptibility profile to beta-lactams, particularly third-generation cephalosporins, which therefore represent a reasonable choice, possibly in combination with clindamycin. CoNS represents an exception, although we cannot exclude the possibility of contamination in these cases.

A systematic review evaluating the effectiveness of pneumococcal vaccines (including 7-, 10-, and 13-valent pneumococcal conjugate vaccines and the 23-valent polysaccharide vaccine) on AOM in children under five years of age demonstrated a significant effect of pneumococcal vaccination on reducing the incidence of AOM in this age group [43].

Some studies have reported a decrease in pneumococcal AM following vaccine introduction [44,45]. Other studies have reported no significant changes, probably due to possible pneumococcal serotype replacement [46].

In our study, the limited number of cultures performed, particularly those that tested positive for *Streptococcus pneumoniae*, along with the lack of reliable data on pneumococcal vaccination coverage, prevented us from accurately assessing the impact of vaccination on the prevention of AM. Pneumococcal vaccination, although not fully documented in the dataset, may play a role in modulating the incidence and severity of AM, an aspect that warrants further investigation.

In our study, the increased use of NSAIDs in the post-COVID-19 period, although not associated with a greater risk of complications, suggests changes in home symptom management strategies, which could influence the timing of hospital presentation and the progression of the disease. NSAIDs have been proposed as potential risk factors for severe progression of bacterial infections, although the underlying biological mechanisms remain controversial. In vitro evidence indicates that NSAIDs reduce C5a- and CXCL8-induced neutrophil migration and F-actin polymerization, independent of COX inhibition and PGE2 release [47]. Several studies have demonstrated that certain NSAIDs inhibit neutrophil adhesion to the human endothelium without altering CD11b/CD18 expression [48] or by triggering L-selectin shedding through NADPH oxidase-dependent generation of superoxide anions at the plasma membrane [49]. Low doses of NSAIDs, such as those used for antipyretic purposes, may paradoxically exert a proinflammatory effect by promoting neutrophil recruitment. Furthermore, NSAIDs use may delay effective treatment by masking early inflammatory signs of bacterial infection. In a case–control study of adult patients with severe sepsis from community-acquired infections, it was shown that NSAIDs use was not significantly more frequent in patients with severe sepsis compared to controls. However, the median time to initiation of effective antibiotic therapy was longer in patients who had used NSAIDs (6 days vs. 3 days) suggesting that NSAIDs use may delay antibiotic treatment, possibly by masking or attenuating early symptoms [50]. A review of studies in both adult and pediatric patients with community-acquired pneumonia identified NSAIDs use as an independent risk factor for pleural empyema, even in studies accounting for protopathic bias. One proposed explanation, the “temporal hypothesis”, suggests that pre-hospital NSAIDs use to relieve pain or fever may mask early symptoms, delaying both diagnosis and initiation of appropriate antibiotic therapy. This delay can lead to a more invasive disease course, with higher rates of pleural empyema and bacteremia [51].

As proposed by previous authors, NSAIDs can modulate host inflammatory pathways and innate immune responses, potentially contributing to the development of severe group A streptococcal infections by increasing the production of cytokines, including TNF, IL-1, and IL-6 [52]. Clinical associations have been reported between NSAIDs use and necrotizing fasciitis during primary varicella [53]. Additionally, an increased risk of pulmonary empyema and sinusitis with intracranial complications following NSAIDs use for viral infections has been described in the literature [54,55].

Analogously, it is plausible to hypothesize a potential link between NSAIDs use and an increased risk of developing IC. However, further studies on larger cohorts, specifying the duration and dosage of NSAIDs use, are needed to confirm this association.

In our case series, CRP levels were significantly elevated in patients with complicated AM compared with those with uncomplicated AM, underscoring its role as a marker of disease severity. Similarly, procalcitonin (PCT) positivity with a cutoff value of >0.5 ng/mL was significantly more common among complicated patients, indicating its potential utility in distinguishing disease severity. However, when focusing exclusively on patients with positive PCT results, the quantitative comparison of PCT levels between complicated and uncomplicated cases did not reveal a statistically significant difference. This loss of significance may be attributed to the reduced sample size in this subgroup analysis, which decreases the statistical power and limits the ability to detect differences, even if they exist.

From a therapeutic standpoint, antibiotic treatment was widely adopted in both periods, with a preference for ceftriaxone, often in combination with other agents to ensure broad-spectrum coverage. Both the duration of antibiotic therapy and the length of hospital stay were significantly longer in the post-COVID-19 period and among complicated cases, partly reflecting the increase in ICs requiring prolonged antibiotic treatment [56]. The increase in hospital admissions and complex complications, which often require surgical procedures and multidisciplinary care, has led to a significant increase in associated healthcare expenditures.

The proportion of surgical interventions increased in parallel with the increase in cases, although the distribution of procedure types remained consistent between the pre- and post-COVID-19 periods. This suggests that despite the higher frequency of admissions and complications, our surgical strategies remained stable, although they required greater use of hospital resources. In our case series, a conservative approach was adopted for uncomplicated cases, consisting of antibiotics combined with minor surgical procedures such as myringotomy with ventilation tube insertion or drainage of the subperiosteal abscess via a retroauricular incision or needle aspiration; mastoidectomy was a second-line procedure.

Specifically, myringotomies were performed in children who presented with consistent retroauricular swelling and bulging of the tympanic membrane.

Mastoidectomy was performed in cases of ICs, either after the neurosurgical procedure or during the same neurosurgical session, for exteriorized mastoid abscesses, cholesteatoma, purulent otorrhea or granulation tissue resistant to systemic antibiotics. In two cases of subperiosteal abscess without ICs, a retroauricular puncture with grommet tube insertion, combined with antibiotic therapy, was chosen over mastoidectomy.

The most frequent indication for craniotomy was the presence of epidural pus collection that could not be drained during the mastoidectomy procedure. In the few cases in which the infection extended beyond the dura, forming a subdural empyema, immediate craniotomy and evacuation were mandatory because of the risk of rapid spread of pus along the subdural space. For intracerebral abscesses, neurosurgical treatment (burr hole aspiration or craniotomy) was performed to drain abscesses larger than 2.5 cm in diameter.

External cerebrospinal fluid (CSF) drainage was used in complicated cases of mastoiditis when intracranial hypertension or hydrocephalus developed secondary to meningitis, cerebritis, or abscess compression or when CSF leakage occurred after surgery to facilitate closure of the dural defect. Temporary external drainage, in fact, helps reduce intracranial pressure, facilitates infection control, and protects surgical repairs of dural defects.

In a recent review of AM management, the success rate of antibiotics alone was 24.6%; overall, the success rate of minor surgical procedures, excluding mastoidectomy, was 87.7%, whereas the success rate of mastoidectomy was 97% [57].

The increased frequency of complicated cases of AM, previously considered rare, has raised clinical concerns among general pediatricians and emergency department clinicians, particularly regarding the appropriate use of radiologic imaging. The diagnosis of AM remains primarily clinical, and imaging studies should not be employed for diagnostic confirmation but rather to evaluate potential complications; this approach is especially critical given the risks associated with radiation exposure, particularly in younger children.

In our case series, CT and/or MRI were performed in a high percentage of patients. When the different periods were compared, no substantial changes in the neuroimaging strategy were observed. Although MRI was used more frequently in the post–COVID-19 period, this increase was not statistically significant and was likely related to the higher incidence of IC. As reported in the literature, the routine use of CT scans, often with contrast enhancement, to detect thrombosis in the majority of pediatric patients diagnosed with AM remains a challenging clinical decision, especially in the presence of complications but the absence of clearly suggestive symptoms [41]. Recently, a study was published that proposed stratification of patients with AM who should undergo radiological evaluation [13].

SARS-CoV-2 has been implicated in AOM, and cases of effusive otitis media have been reported in which SARS-CoV-2 was detected in drained middle ear fluid via real-time reverse-transcriptase polymerase chain reaction (RT-PCR) [58]. A retrospective study in adult patients with acute neurological symptoms related to COVID-19 infection but without respiratory manifestations demonstrated that, despite a high viral load in the nasal cavity and nasopharynx, there was limited evidence of disease in the sinuses, nasopharynx, or mastoid cavity. CT imaging revealed mastoid opacification in only 7% of patients [59]. Another retrospective study of 83 adult patients with SARS-CoV-2 infection reported that paranasal sinus opacification was present in 51.8% of patients, whereas middle ear or mastoid opacification was observed in only 24.1% of patients [60]. A case involving an adult patient with mastoiditis complicated by subdural empyema has also been reported. Although the patient had an acute SARS-CoV-2 infection, intraoperative cultures revealed *Streptococcus pyogenes*, and RT-PCR testing was negative for SARS-CoV-2 [61]. Several studies have investigated nasopharyngeal microbiome alterations and disease susceptibility in patients following SARS-CoV-2 infection [62,63]. However, particularly during the post-COVID-19 period, few studies have demonstrated a correlation between the increased incidence of AM, especially its complicated forms, and recent or prior SARS-CoV-2 infection, as patient infection status has not been systematically assessed through serological testing or RT-PCR.

### Limitations

Our study has several limitations that should be taken into account when the findings are interpreted. First, the retrospective design implies reliance on the completeness and accuracy of medical records, which introduces the risk of information bias and limits the ability to control for confounding variables. The incomplete availability of certain data, such as vaccination status and prehospital use of antibiotics or NSAIDs, reduced the robustness of some analyses and may have introduced bias in subgroup comparisons. As this is a single-center study conducted in a tertiary-level pediatric hospital, albeit with a large sample size, the generalizability of the findings to other healthcare settings, particularly primary care facilities or institutions with different organizational models, may be limited. Importantly, the microbiological data were limited to patients who underwent surgical procedures, limiting the ability to assess the overall pathogenic flora and the potential emergence of antimicrobial resistance.

## 5. Conclusions

To our knowledge, this is one of the few European single-center studies with a large case series to provide a detailed temporal, clinical, and microbiological analysis of AM in children before and after the pandemic.

Our study demonstrated that the COVID-19 pandemic has significantly influenced the epidemiology and clinical severity of pediatric AM. The increase in complications and longer hospital stays demand careful planning of healthcare resources and reinforcement of prevention and early diagnosis strategies to limit the clinical and socioeconomic impact of this condition.

These findings highlight the importance of effective preventive strategies, including enhanced vaccination coverage, the promotion of early diagnosis, and the optimization of outpatient therapy to reduce the risk of complications and, consequently, the associated costs. Additionally, implementing standardized clinical protocols could support more efficient and consistent management, reducing hospital stays and recurrence rates.

The COVID-19 pandemic has served as a real-world model, illustrating how the course of endemic infectious diseases, such as AM, can be dramatically altered by public health interventions implemented in response to a viral epidemic.

## Figures and Tables

**Table 1:** Absolute and relative frequencies of presenting symptoms of AM in the pre-COVID-19 and post-COVID-19 periods.

Symptoms	Pre-COVID-19 No. (%)	Post-COVID-19 No. (%)	*p*
Mastoid swelling	50 (71.4%)	129 (69.4%)	*0.75*
Headache	8 (11.4%)	89 (47.9%)	*<0.01*
Fever	38 (54.3%)	158 (85.0%)	*<0.01*
Vomiting	6 (8.6%)	40 (21.5%)	*<0.05*
Otalgia	55 (78.6%)	160 (86.0%)	*0.15*
Seizures	1 (1.4%)	13 (7.0%)	*0.12*
Altered consciousness	0 (0%)	17 (9.1%)	*<0.01*
Photophobia	1 (1.4%)	13 (7.0%)	*0.12*
Cranial nerve deficits	8 (11.4%)	8 (4.3%)	*<0.05*

**Table 2:** (**a**) Absolute and annualized frequencies of extracranial and intracranial complications of AM in the pre-COVID-19 and post-COVID-19 periods. (**b**) Incidence rate ratio of ECs and the IC of AM before and after COVID-19 for complications with ≥10 cases total.

(a)				
Complications	Pre-COVID-19		Post-COVID-19	
	Per Year	Total No.	Per Year	Total No.
**Extracranial complications**	4.4	14	6.7	20
Facial nerve palsy	1.9	6	1.0	3
Bezold’s abscess	0.9	3	0.0	0
Osteomyelitis	1.3	4	1.0	3
Subperiosteal abscess	2.5	8	5.0	15
**Intracranial complications**	3.8	12	12.3	37
Thrombosis	2.8	9	8.7	26
Meningitis	0.6	2	3.0	9
Brain abscess	0.3	1	1.0	3
Epidural empyema	2.2	7	6.0	18
Subdural empyema	0.9	3	2.3	7
**(b)**				
**Complications**	**IRR**		**95% CI**	
**Extracranial complications**	1.51		0.72–3.23	
Subperiosteal abscess	1.98		0.79–5.39	
**Intracranial complications**	3.25		1.66–6.86	
Thrombosis	3.05		1.38–7.39	
Meningitis	4.75		0.98–45.18	
Epidural empyema	2.71		1.08–7.68	
Subdural empyema	2.46		0.56–14.76	

## Data Availability

The original contributions presented in this study are included in the article. Further inquiries can be directed to the corresponding author.

## Abbreviations

AOM	acute otitis media
AM	acute otomastoiditis
CT	computed tomography
MRI	magnetic resonance imaging
CRP	C-reactive protein
IV	intravenous
EC	extracranial complications
IC	intracranial complications
US	United States
NPIs	nonpharmaceutical interventions
NSAIDs	Nonsteroidal Anti-Inflammatory Drugs
PCT	Procalcitonin
IRR	incidence rate ratio
RSV	Respiratory syncytial virus
RT-PCR	real-time reverse-transcriptase polymerase chain reaction
